# Integrative analysis of transcriptome complexity in pig granulosa cells by long-read isoform sequencing

**DOI:** 10.7717/peerj.13446

**Published:** 2022-05-25

**Authors:** Shuxin Li, Jiarui Wang, Jiale Li, Meihong Yue, Chuncheng Liu, Libing Ma, Ying Liu

**Affiliations:** School of Life Science and Technology, Inner Mongolia University of Science & Technology, Baotou, Inner Mongolia, China

**Keywords:** Pig, Granulosa cells, Iso-Seq, LncRNAs, Alternative splicing

## Abstract

**Background:**

In intensive and large-scale farms, abnormal estradiol levels in sows can cause reproductive disorders. The high incidence rate of reproductive disturbance will induce the elimination of productive sows in large quantities, and the poor management will bring great losses to the pig farms. The change in estradiol level has an important effect on follicular development and estrus of sows. To solve this practical problem and improve the productive capacity of sows, it is significant to further clarify the regulatory mechanism of estradiol synthesis in porcine granulosa cells (GCs). The most important function of granulosa cells is to synthesize estradiol. Thus, the studies about the complex transcriptome in porcine GCs are significant. As for precursor-messenger RNAs (pre-mRNAs), their post-transcriptional modification, such as alternative polyadenylation (APA) and alternative splicing (AS), together with long non-coding RNAs (lncRNAs), may regulate the functions of granulosa cells. However, the above modification events and their function are unclear within pig granulosa cells.

**Methods:**

Combined PacBio long-read isoform sequencing (Iso-Seq) was conducted in this work for generating porcine granulosa cells’ transcriptomic data. We discovered new transcripts and possible gene loci via comparison against reference genome. Later, combined Iso-Seq data were adopted to uncover those post-transcriptional modifications such as APA or AS, together with lncRNA within porcine granulosa cells. For confirming that the Iso-Seq data were reliable, we chose four AS genes and analyzed them through RT-PCR.

**Results:**

The present article illustrated that pig GCs had a complex transcriptome, which gave rise to 8,793 APA, 3,465 AS events, 703 candidate new gene loci, as well as 92 lncRNAs. The results of this study revealed the complex transcriptome in pig GCs. It provided a basis for the interpretation of the molecular mechanism in GCs.

## Introduction

In mammals, granulosa cells (GCs) have very important function in oocyte development (Hoffmann & Maser, 2007; Park et al., 2004; Robinson et al., 2012; Yamochi, Hashimoto & Morimoto, 2021). Every follicle includes one GCs-surrounded oocyte, while GCs can function in supporting the oocyte. GCs can regulate follicular development and secondary sexual characteristics of female animals through hormone secretion (McGee & Hsueh, 2000); meanwhile, GCs participate in the growth and atresia of follicles through proliferation and apoptosis, which play a very important role in the reproductive process of female animals (Manabe et al., 2004; Quirk et al., 2004). Therefore, it is significant to investigate the regulatory signaling pathways and the transcriptional/post-transcriptional mechanisms in GCs apoptosis and hormone synthesis. So far, pork is still the main variety of meat consumption of Chinese residents. So pig is an important species in animal husbandry. Improving the fecundity of sows is of great significance to the benefits of pig breeding. The studies about the transcriptional events in pig GCs can provide experimental basis to improve the sow fertility.

The gene function and mechanism of GCs have been illustrated through a variety of research methods. Among them, genome sequencing is the basis of genomic research, and it has been widely used in animal research. The second-generation sequencing is a frequently used method to analyze the difference in gene expression. However, the long reading length of the sequence in second-generation measurement is only 50-500 bp. The short read techniques are associated with some innate drawbacks like difficult discrimination of paralogous sequences, GC bias, difficult repetitive element mapping, as well as difficult allele phasing (Ardui et al., 2018). The long-read mappability accounts for a key factor to confirm repetitive elements, gene fusions and gene isoforms. Fortunately, third-generation sequencing has solved the problems encountered by second-generation technique without repeating element limitation, and its long reading length is more than 20 kb (Eid et al., 2009; Korlach et al., 2010). Second-generation sequencing has been widely used in revealing the regulation of gene expression in GCs (Du et al., 2021; Li et al., 2021b; Toms et al., 2021; Xu et al., 2022), but third-generation sequencing is rarely used. Third generation sequencing can better reveal the phenomenon of complex gene transcription regulation, and provide a basis for analyzing the function of gene regulation in GCs.

Numerous articles are conducted to explore GCs-related gene functions as well as the precise mechanisms. Previous studies have suggested that miRNAs (Tian et al., 2020; Toms et al., 2021), lncRNAs (Hu et al., 2021; Meng et al., 2021; Ruszkowska et al., 2018; Zhang et al., 2019) and mRNAs (Kulus et al., 2021; LaVoie, 2017; Shi & Sirard, 2021) have critical effects on modulating GCs function along with follicular development in pigs. In addition, for precursor-messenger RNAs (pre-mRNAs), their post-transcriptional modifications like alternative polyadenylation (APA) and alternative splicing (AS) enrich the proteome diversity and have critical effect in different tissues and different developmental stages (Baralle & Giudice, 2017; Di Giammartino, Nishida & Manley, 2011; Gruber & Zavolan, 2019). In pigs, previous studies report that AS plays an essential role in estrum of sows and in male fertility (Tang et al., 2018; Zhang et al., 2018). In pig GCs, cellular-FLICE like inhibitory protein (cFLIP) and estrogen receptor beta (ER beta) have two alternative splicing isoforms and participate in follicular development (LaVoie et al., 2002; Matsuda-Minehata et al., 2005). APA is associated with the rapid and slow porcine muscle development (Deng et al., 2020). In a word, there are few reports on the occurrence and function of gene AS and APA in GCs and the follicular development of pigs.

The PacBio Isoform sequencing technology (Iso-Seq) is an effective method for the integrative analysis of transcriptome complexity for revealing gene functions as well as mechanisms. This study employed PacBio third-generation sequencing for illustrating post-transcriptional modifications of Yorkshire pig GCs. Besides, reverse transcription PCR (RT-PCR) was adopted in combination to investigate different spliceosomes of genes. According to our results, GCs of pig showed transcriptome complexity, which could serve as the basis to reveal the mechanism of follicular development and the candidate DNA marker in porcine marker-assisted selection (MAS).

## Materials & Methods

### Ovary collection

Each animal experiment was conducted according to China Council on Animal Care guidelines. Our study protocols gained approval from NMGKJDX Laboratory Animal Management Committee (NMGKJDX-2019-10). We collected and kept adult Yorkshire pig ovaries within sterile normal saline (0.9% NaCl, 1% penicillin-streptomycin) under 37 °C at the local slaughterhouse. Then the ovaries were delivered to the laboratory as soon as possible.

### GCs cell culture

To culture cells, we used a 20 ml syringe to aspirate follicular fluid in 3–5 mm follicles, which was later subject to 5 min centrifugation at 1,000 g prior to GCs collection. We later rinsed GCs thrice by serum-free DMEM/F12 medium (Invitrogen, Waltham, MA, USA), dispersed and inoculated them within the six cm plates at 1 ×10^6^/well with DMEM/F12 (5 ml) that contained 10% fetal bovine serum (Invitrogen, Waltham, MA, USA). Thereafter, we cultivated cells under 37 °C and 5% CO_2_ conditions for 72 h period. We replaced the medium at 24 h intervals (Yu et al., 2016).

### Transcriptome library preparation and sequencing

We utilized TRIzol reagent (Invitrogen, Waltham, MA, USA) to extract total cellular RNA in line with specific protocols. After purification, we adopted DNase I (Qiagen, Hilden, Germany) for enzymatic digestion. Later, we utilized the Agilent 2100 Bioanalyzer (Agilent Technologies, Santa Clara, CA, USA) to assess RNA purity and content. Meanwhile, we adopted SMARTer PCR cDNA Synthesis Kit (Takara Biotechnology, Shiga, Japan) to generate cDNA and constructed libraries with SMRTbell™ Template Prep Kit 1.0 (Pacific Biosciences, Menlo Park, CA, USA). Subsequently, we adopted Sequel™ Sequencing Kit 2.0 (Pacific Biosciences) for library sequencing (Shanghai Personalbio Technology Co., Ltd., Shanghai, China).

### Iso-Seq data processing

**Figure 1:**
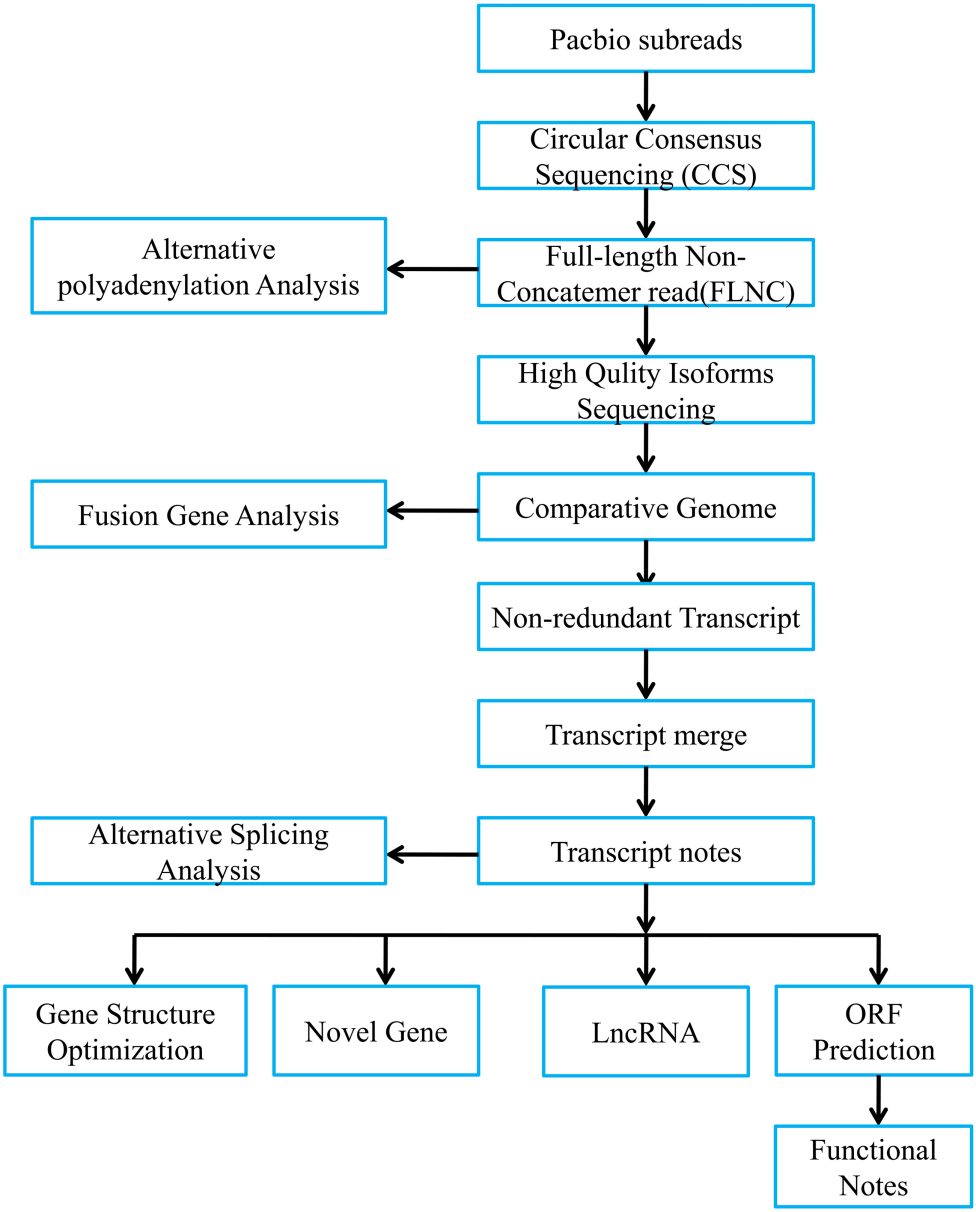
Overview of bioinformatic pipeline.

Figure 1 presented bioinformatic analysis pipeline. We employed SMRT Link v8.0 (https://www.pacb.com/wp-content/uploads/SMRT-Link-User-Guide-v8.0.pdf) to preprocess those Iso-Seq raw reads. Then, we obtained subreads through removing polymerase read adapters. With the parameters below, we acquired circular consensus sequencing (CCS) reads based on those subreads, which were maximum/minimum subread length = 15,000/50, minimum prediction accuracy = 0.99 and minimum pass number = 3. Afterwards, we adopted lima software to divide CCS as full-length reads. Typically, full-length CCS reads that contained the 3′ and 5′ polyA and cDNA primers were deemed as full-length non-chimeric reads (FLNCs). Using default parameters, we conducted FLNC correction of high-quality reads through LoRDEC v0.9 software. Iso-seq data were examined using SQANTI2 (Tardaguila et al., 2018). We finally utilized GMAP to align FLNCs into pig reference genome (https://asia.ensembl.org/Sus_scrofa_largewhite/Location/Genome?db=Core).

### Identification of lncRNAs

This study discovered possible lncRNAs from porcine GCs according to our prior work (Yang et al., 2017). To be specific, we first eliminated short (<200 nt) transcripts, single-exons, as well as transcripts overlapped with those constructed gene models of consensus transcriptome. Secondly, we adopted 4 programs, namely, CPC (v0.9-r2) (Kang et al., 2017), CNCI (v2) (Sun et al., 2013), PLEK v1.2 (minlength−200) (Li, Zhang & Zhou, 2014) as well as CPAT v1.2.4 (default parameters)(Wang et al., 2013) for predicting new isoforms-based lncRNAs.

### Isolation of RNA and reverse transcription PCR (RT-PCR)

We treated 1 µg total RNA after purification to be the template to synthesize cDNA by adopting Moloney murine leukemia virus (M-MLV, Promega, USA). Non-template controls were included in each RT-PCR. We adopted Bio-rad T100 Thermo Cycler (Bio-rad, Hercules, CA, USA) to conduct RT-PCR with a Taq PCR Mix (TaKaRa, Japan) in a 30 µl reaction system. The reaction conditions were shown below, 5 min under 95 °C; 35 s under 95 °C, 30 s under 58 °C, 40 s under 72 °C for 35 cycles; and final 40 s under 72 °C. Primers utilized in the present work were prepared by Primer 5.0, as shown in Table S1.

## Results

### PacBio Iso-Seq and bioinformatic analysis

After quality control, 385,343 CCS reads (mean depth, 39.68 passes) and 1,027,326,158 filtered subreads (average length, 2,666 bp) were generated (Table 1). After insertion fragment recognition, sequences were classified as three types: non-full length sequence (nFL_reads), full-length chimeric sequences (FL_chimera_reads) and full-length non-chimeric sequences (FL_non-chimera_reads, FLNCs). The latter included full-length non-chimeric sequences with PolyA (FLNC_withPolyA_reads) and full-length non-chimeric sequences without PolyA (FLNC_withoutPolyA_reads). The read classification results were shown in Table 2. Finally, there were only 301,082 FLNCs used for subsequent analysis. FLNCs sequence was clustered and corrected by isoseq3 cluster software to obtain the high quality (HQ) isoform (Table 3). Subsequent analysis was conducted based on the HQ isoform.

**Table 1:** Summarized information of Iso sequencing (Iso-Seq) data.

**Sample**	**A1**
Reads of Insert	385,343
Read Bases of Insert	1,027,326,158
Mean Read Length of Insert	2,666
Mean Read Quality of Insert	1
Mean Number of Passes	39.68

**Table 2:** Reads of insert (ROI) classification statistics.

**Classification**	**A1**
Total_ccs	385,343
nFL_reads	83,203
FL_chimera_reads	1,058
FLNCs	301,082
FLNC_withoutPolyA_reads	570
FLNC_withPolyA_reads	300,512

**Table 3:** HQ isoforms by isoseq3.

**Sample**	**A1**
High quality isoforms	21,863
High quality isoforms total bases	59,311,425

### Gene loci and isoform detection

**Figure 2:**
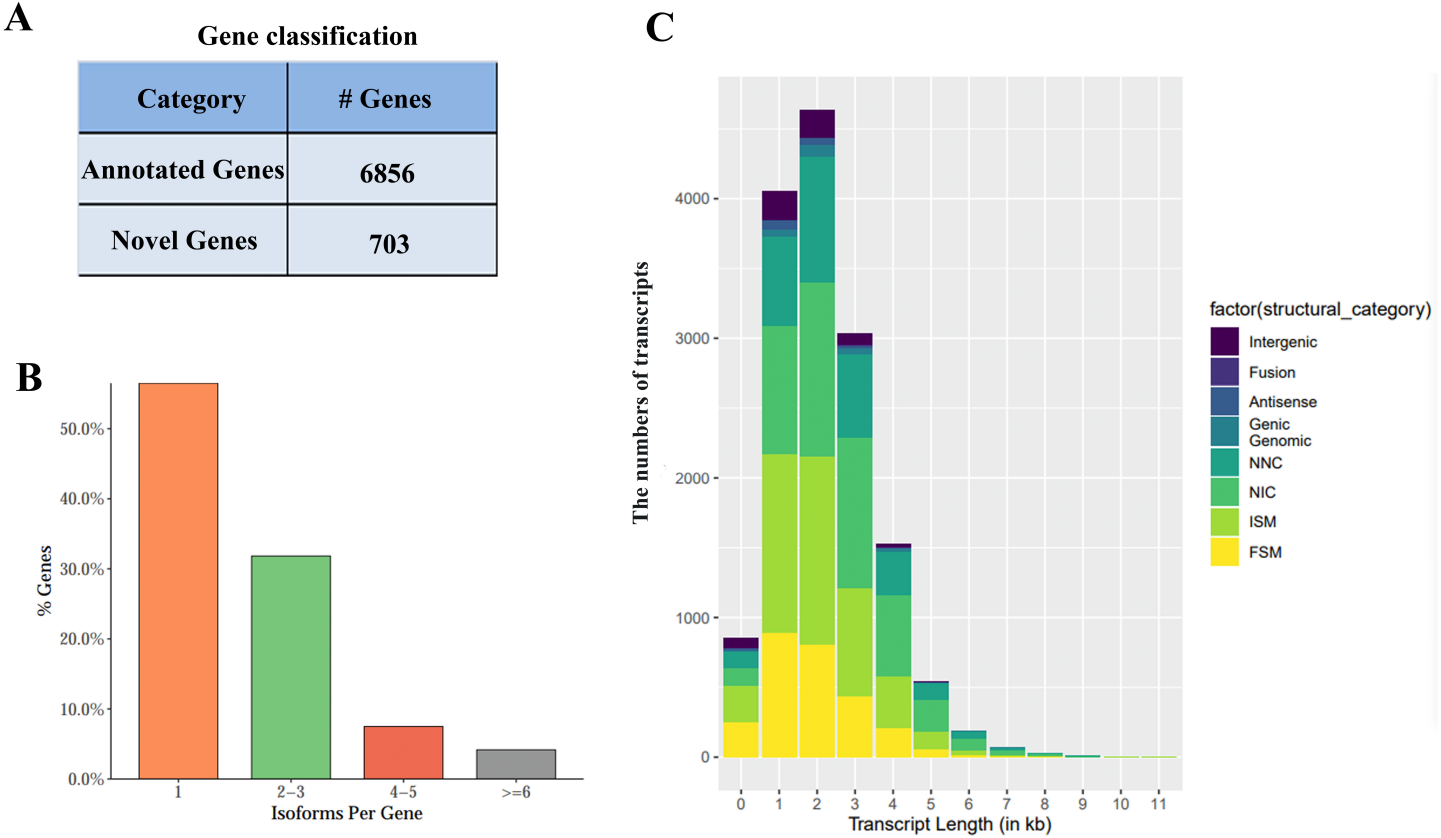
Iso-sequencing (Iso-Seq) data-based identification of genes together with isoforms. (A) Gene classification. (B) Transcript distribution. (C) Classifications by isoforms length.

FLNC sequence was corrected to analyze the alignment position as well as sequencing errors. Based on FLNC alignment position, each gene locus and isoform was detected. Altogether 7,559 gene loci (including 703 possible new and 6,856 identified ones) were discovered using Iso-Seq (Fig. 2A). About half of the genes had more than one splice (Fig. 2B). Those new gene loci as well as new isoforms were added into the reference annotation file (Table S2).

Isoforms were compared with the information of known transcripts according to the position and cutting information relative to the genome using SQANTI2. Isoforms were divided into FSM (full splice match), ISM (incomplete splice match), NIC (Novel in Catalog), NNC (Novel Not in Catalog), Antisense, Genic, Genomic, Fusion, and Intergenic subtypes. The length distribution of different types of isoforms was shown in Fig. 2C.

### LncRNA prediction

**Figure 3:**
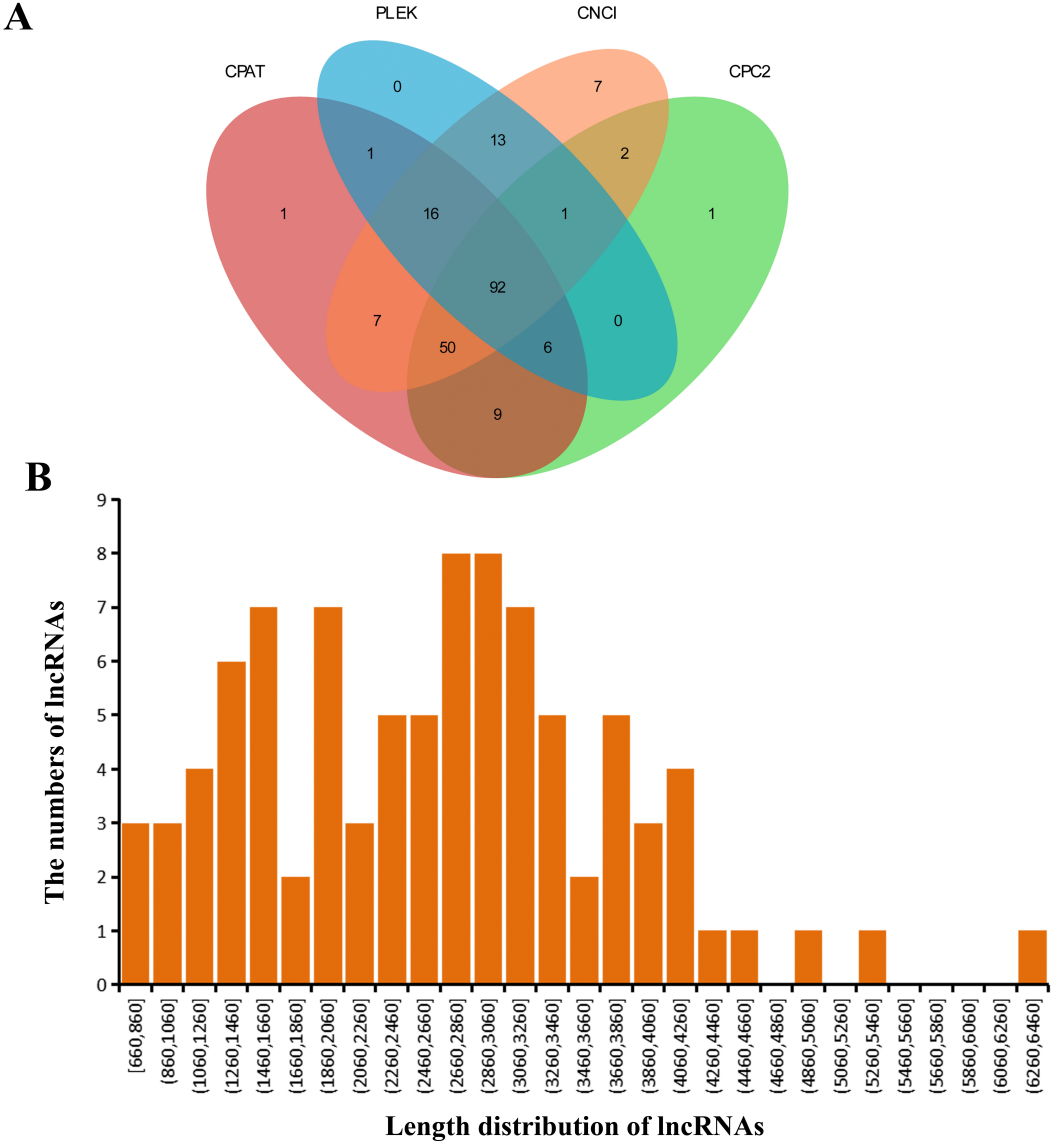
Detected lncRNAs. (A) Venn plot showing those lncRNAs detected *via* four software applications. (B) Predicted lncRNA lengths.

Four softwares were adopted for lncRNA prediction in the GCs transcriptome. At last, we estimated 92 lncRNAs in total (Fig. 3A). Table S3 listed the predicted lncRNA sequences, whose lengths were 660-6,460 bp (average, 2,623 bp) (Fig. 3B).

### Alternative polyadenylation events detection

**Figure 4:**
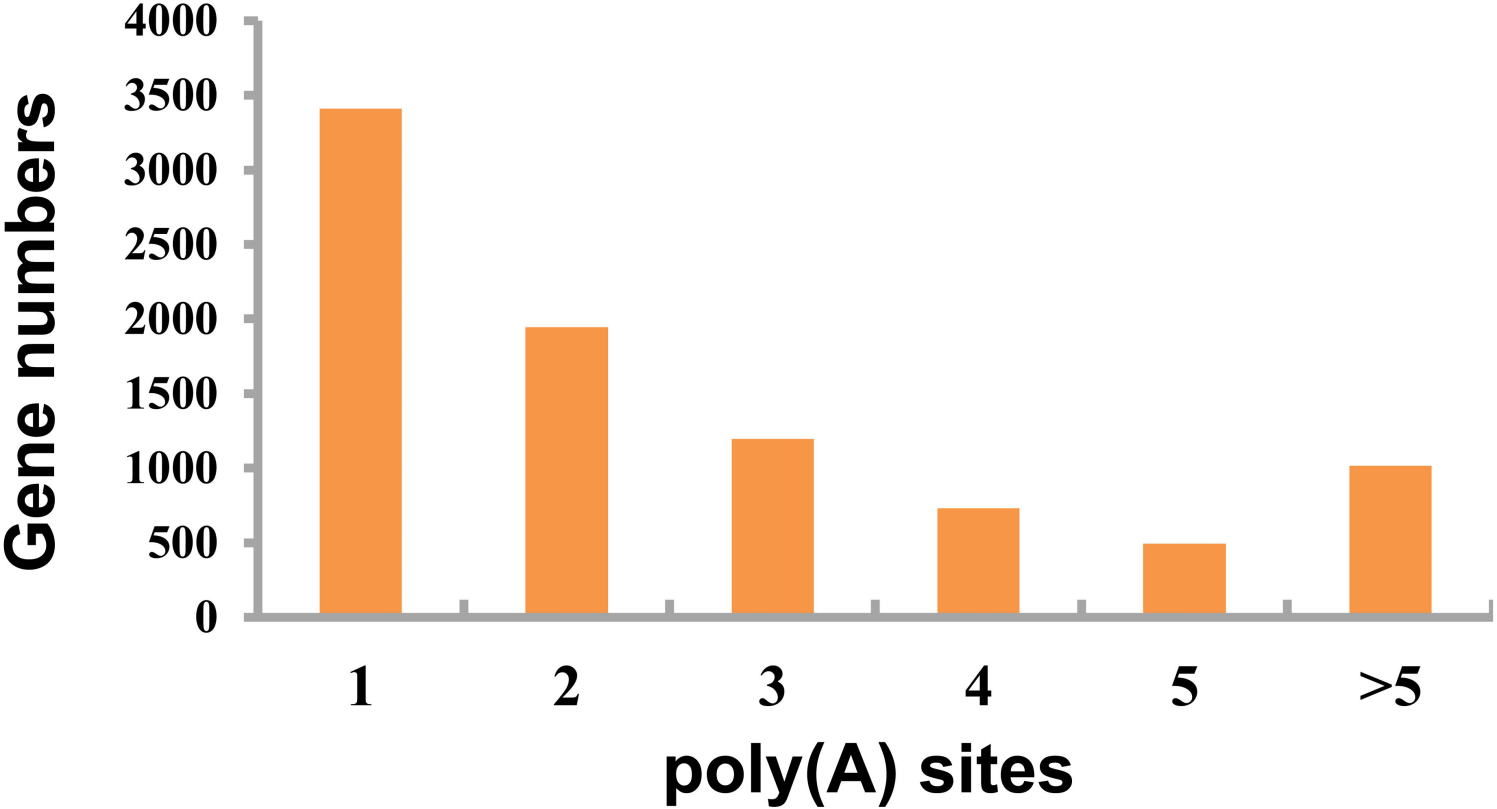
Detected APA events. Distribution of different numbers of poly(A) sites.

This study detected APA events within porcine GCs using TAPIS pipeline. Among those genes identified 8,793 showed one or more poly(A) site (Fig. 4, Table S4), including 3,412 containing one single poly(A) site (Fig. 4), whereas 5,381 (61.20%) containing at least two poly(A) sites (Fig. 4).

### Alternative splicing events detection

**Figure 5:**
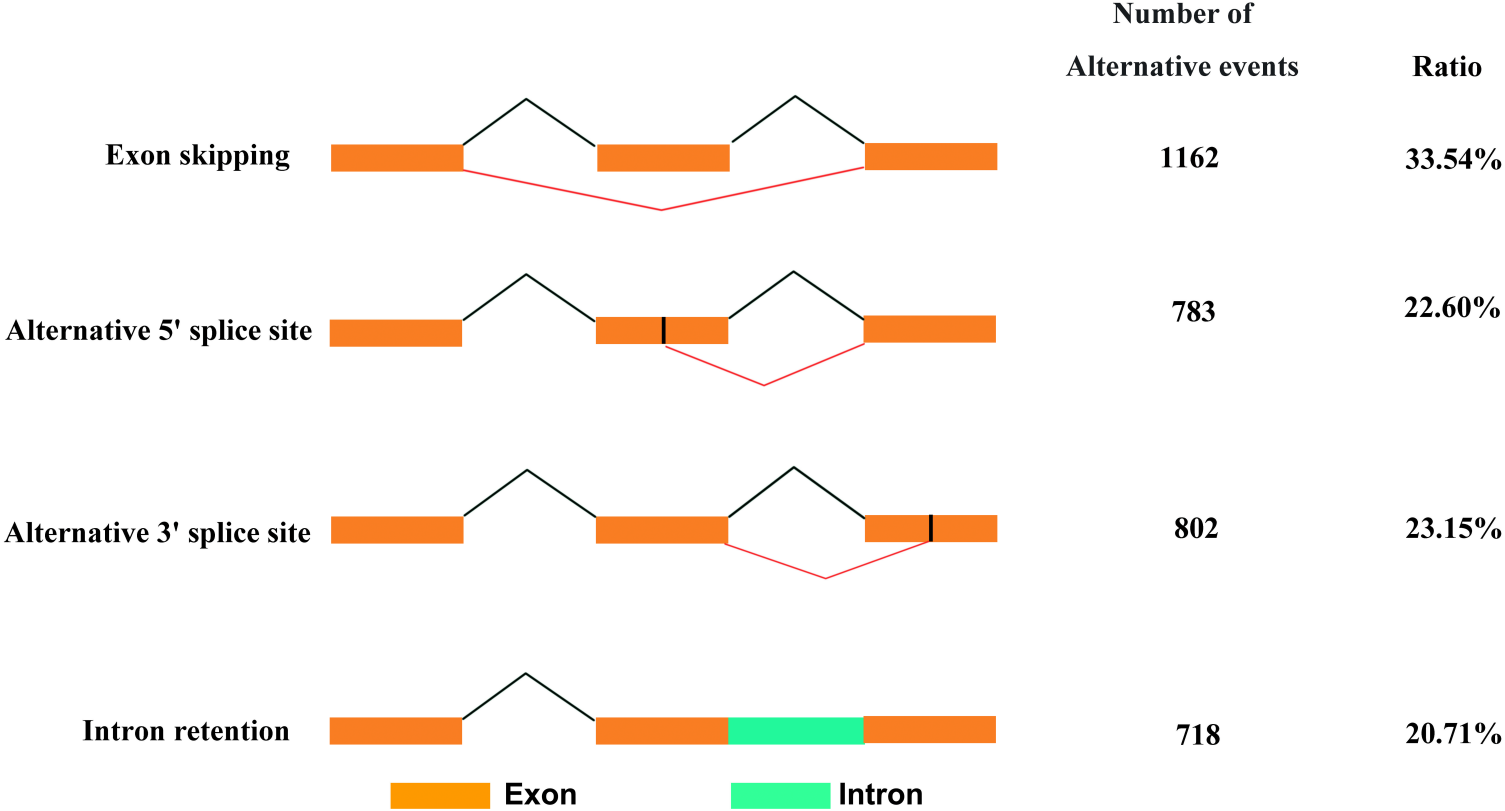
Classification of AS events.

Based on Iso-Seq data, we identified 3,465 AS events in total. There were four types of AS events (Fig. 5), among which, exon skipping (1,162, 33.54%) showed the highest prevalence, while alternative 5′ (783, 22.60%), alternative 3′ (802, 23.15%) and intronretaining (718, 20.71%) ranked the second to fourth places, respectively.

### Validation of alternative splicing genes

**Figure 6:**
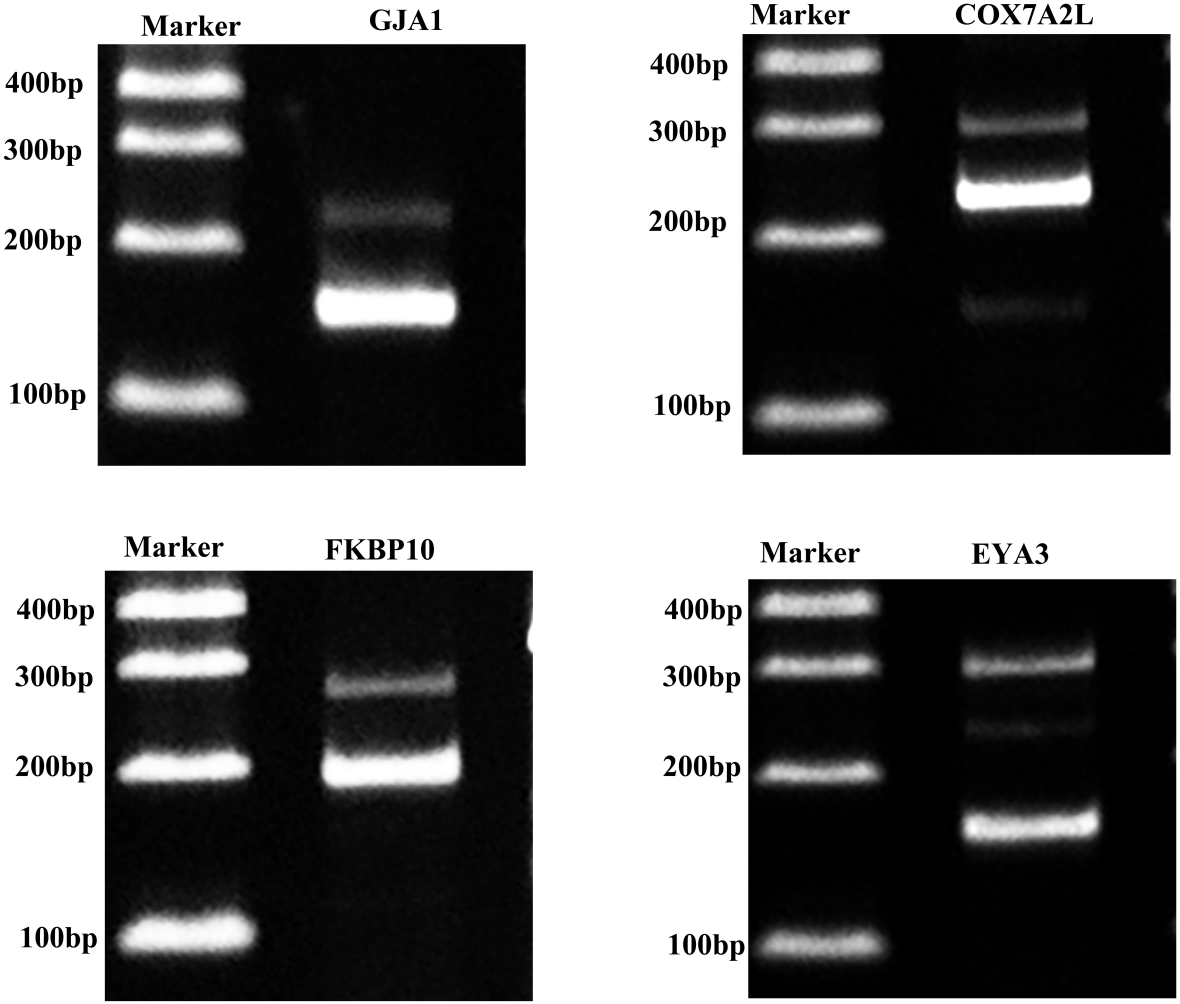
Validation of AS events.

For confirming whether the Iso-Seq results were reliable, we screened four AS genes within porcine GCs through RT-PCR (Fig. 6). RT-PCR came to similar results to sequencing results, which indicated that the Iso-Seq data were reliable.

### Fusion gene analysis

Gene fusion refers to the process where all or part of the sequences of two genes fuse with each other to form a new gene. The production of fusion gene may be the consequence of chromosome translocation, intermediate deletion or chromosome inversion, which is usually tumorigenic. Gene fusion is a common feature of tumor, which enhances cancer genesis and progression, and is adopted to be the diagnostic and therapeutic target against tumors. Using Fusion Finder, multiple genes were extracted in the alignment results, and the sequences with certain reads support were the candidate fusion genes. Later, 147 candidate fusion genes were annotated. The annotation results were displayed in Table 4 and Table S5.

**Table 4:** Fusion gene annotation results (example).

Isoform	Position	Part1_transid	Part2_transid
PBfusio n.64	LUXX01048117.1:2433701-24 33871:-—LUXX01048117.1:298 9215-2989242:-	ENSSSCG00025040040_ENSSSC G00025039201	ENSSSCG00025040040
PBfusio n.113	LUXX01039762.1:1602380-16 02480:-—LUXX01052712.1:101 603-101739:-	ENSSSCG00025022795	ENSSSCG00025056743
PBfusio n.52	LUXX01021592.1:400524-400 768:+—LUXX01021592.1:72958 8-729843:+	ENSSSCG00025024992	ENSSSCG00025024992

### Cluster analysis of isomers by GO

**Figure 7:**
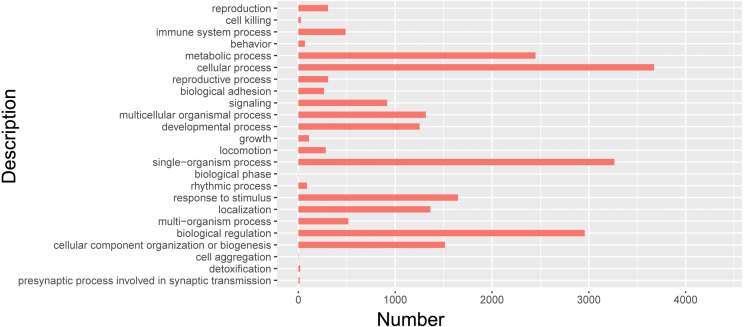
GO biological analysis of isoforms in pig GCs.

In order to further clarify the biological function of isoforms from Iso-Seq, cluster analysis was carried out by using GO. GO databases showed isoforms participated in 24 biological processes (Fig. 7, Table S6). Among them, 712 isoforms were related to reproductive process. The most isoforms were enriched in cellular process. Detailed information of other biological processes was shown in Table S6.

## Discussion

The third generation sequencing technology is under rapid development in recent years, which has been applied to numerous research fields, such as genome resequencing (Li et al., 2016), *de-novo* genome assembly (Jenjaroenpun et al., 2018; Morabito et al., 2020), transcriptome research (Mannarapu, Dariya & Bandapalli, 2021; Yuan et al., 2021), methylation detection (Liu et al., 2019a; Pillai, Gopalan & Lam, 2017), disease-related structural variation detection (Cretu Stancu et al., 2017; Roberts et al., 2021), and virus analysis in epidemiology.

Previously, the second-generation sequencing was the most commonly used method to analyze gene molecular mechanism in pig (Liu et al., 2019b; Tang et al., 2017). Recently, third-generation sequencing has been increasingly employed in studying the functional genomics and epigenetics in pig (Beiki et al., 2019; Ma et al., 2021; Zhang et al., 2020). However, the AS and APA events in the pig ovary are rarely reported. In the present study, we revealed the transcriptomic complexity in porcine GCs using PacBio long-read sequencing, resulting in 8,793 APA, 3,465 AS events, 703 candidate new gene loci, together with 92 lncRNAs.

LncRNAs can modulate gene levels within different biological events. Some studies have conducted to identify lncRNA functions within porcine GCs and ovary. Liu et al. (2018) identified altogether 2076 lncRNAs (including 714 novel and 1,362 known ones) from the libraries established based on Duroc ovaries. Ruszkowska et al. (2018) discovered 1,666 lncRNAs within porcine GCs. Knapczyk-Stwora et al. (2019) found altogether 5,592 RNA sequences to be lncRNAs, among which, 136 (at 53 long non-coding loci) were annotated within relevant databases among 11-day-old piglet ovaries. Meanwhile, the same authors discovered altogether 4,669 RNA sequences to be lncRNAs, among which, 1,236 (at 355 long non-coding loci) were annotated within relevant data-bases in pigs by illumina sequencing (Knapczyk-Stwora et al., 2020). In the current study, we found 92 lncRNAs from porcine GCs by PacBio sequencing, and such number was significantly lower than previously reported. We speculated that the reason might be that the third-generation sequencing method was more accurate in analyzing lncRNAs than the second-generation sequencing.

APA has certain influence on 3′-untranslated region (3′-UTR) composition and length, which also modulates the translation or stability of mRNA for affecting vital biological events. Wu and colleagues discovered the dynamic alterations in 3′-UTR landscape when oocytes became mature, which might be related to the modulation of porcine oocyte meiosis (Wu et al., 2021). As reported by Deng et al. (2020) APA had a certain effect on the rapid and slow muscle growth regulated by miRNAs as well as RNA binding proteins (RBPs). Polyadenylation site (PAS) is possibly related to immune response as well as androstenone contents within pigs (Wang et al., 2016). In this study, result showed that Npr3 (ENSSSCG00025035065) had 5 APA sites and could generate different mRNA isoforms in pig GCs. Previous reports detected luteinizing hormone could regulate the expression of Npr3 in mouse and bovine GCs (Dos Santos et al., 2018; Lee et al., 2013). However the function of different mRNA isoforms of Npr3 is needed to further research. The genes which were similar to Npr3 with multiple APA sites in porcine GCs, we found in total 5,381 genes. The APA sites of these genes greatly enriched the diversity of proteins in porcine GCs and provided favorable conditions for the function of GCs.

RNA splicing is a form of post-transcriptional modification, and gene AS is common in eukaryotes. With the application of new generation sequencing technology, recent studies on AS of porcine ovary suggest that 94.4–95.5% of the expressed genes in pig ovary are selectively spliced, and the frequency of AS events is similar in estrous and intersexual periods. Functional analysis of genes with AS events finds that many genes related to hormone metabolism and gonadal development are under different levels of modulation (Tang et al., 2018). In addition, the relationship between steroid hormones and AS events has gradually attracted the attention of researchers. Studies have shown that estradiol modulates class B scavenger receptor (sr-b) AS within liver cells by acting on the AS factors (Zhang et al., 2007). AS is closely related to emotional regulation in postmenopausal women (Hou et al., 2018). Different splice isomers of ERβ are linked with estradiol and RBP (Shults et al., 2018). Studies have shown that the splice isomer of estrogen receptor ER-α 36 could promote the activity of tamoxifen agonist in glioblastoma cells (Qu et al., 2019). Previous studies showed that GJA1 was expressed in GCs and related to the proliferation of GCs in rat, human and bovine (Chen et al., 2013; Lin et al., 2021; Rovani et al., 2014). In this study the result showed that GJA1 had two different isoforms in pig GCs. However, whether their functions in the pig were similar with other species needed further research. FKBP10 could promote proliferation of glioma cells (Cai et al., 2021). The function and expression of FKBP10 had not been reported in GCs. The results of this study demonstrated that it expressed in GCs and had different splicing isomers. C1QTNF3 was revealed to have different transcripts in pig GCs in this study and also was reported to promote proliferation of granulosa cells and protect of granulosa cells from apoptosis in mouse and human (Gershon & Dekel, 2020). The previous reports almost are about AS, estradiol and related diseases or the function of genes. However, there are rarely reports regarding AS occurrence as well as the regulatory and action mechanisms of different splices in porcine GCs. Our results found altogether 3,465 AS events by Iso-Seq data. Such results were more accurate than the sequencing results of ovarian tissue, which could provide a basis for revealing the physiological functions such as steroid hormone synthesis in GCs.

Gene fusion can modify epigenetically, and the resultant products-encoded new proteins are related to carcinogenesis. But little research is conducted to analyze chimeric genes. Li and colleagues examined chimeric genes for their biological effects as well as related mechanisms by weighted co-expression network analysis (Li, Li & Ma, 2021a). Liu et al. (2021) firstly reported that the chimeric RNAs related mechanism in modulating skeletal muscle development in pigs. This work analyzed altogether 147 fusion genes in porcine GCs. This study offered a new direction for exploring chimeric genes’ effect on GCs.

## Conclusions

We employed PacBio Iso-Seq in this work to generate the integrative transcriptomic data for porcine GCs, which gave rise to 8,793 APA, 3,465 AS events, 703 possible new gene loci, together with 92 lncRNAs. This work illustrated that porcine GCs had a complex transcriptome and discovered numerous possible transcripts, thus facilitating to understand follicular development.

## Supplemental Information

10.7717/peerj.13446/supp-1 Supplemental Information 1 Primers of genes

10.7717/peerj.13446/supp-2 Supplemental Information 2 New gene loci and new isoforms

10.7717/peerj.13446/supp-3 Supplemental Information 3 Predicted lncRNA

10.7717/peerj.13446/supp-4 Supplemental Information 4 Alternative polyadenylation events

10.7717/peerj.13446/supp-5 Supplemental Information 5 Fusion gene

10.7717/peerj.13446/supp-6 Supplemental Information 6 GO biological analysis of isoforms

10.7717/peerj.13446/supp-7 Supplemental Information 7 RT-PCR raw data

## Additional Information and Declarations

### Data Availability

The following information was supplied regarding data availability: The sequences are available at NCBI Sequence Read Archive: SRR17818022.
