# Supplementary material for: Integrative analysis of transcriptome complexity in pig granulosa cells by long-read isoform sequencing
Source: PeerJ. 2022 May 25;10:e13446. doi: 10.7717/peerj.13446 (PMC9147391; doi:10.7717/peerj.13446)
Supplement: Supplemental Information 1 [file peerj-10-13446-s001.docx]

**Table S1. Primers of genes**

| Gene | Primer sequence (5′-3′) | Alternative splicing Size (bp) |
| --- | --- | --- |
| GJA1 | Forward: GTCTGAGTGCCTGAACTTGC  Reverse: GCGGTGGAATAGGCTTGAAC | 153  224 |
| EYA3 | Forward: GCTTATCCTGGGCAGACTCA  Reverse: CTGCTGCTGGAATATTGGCA | 160  298 |
| COX7A2L | Forward: CAGGGATTAAGGCCTCTGGT  Reverse: CAGGCAGTAGATGGTCCCTC | 237  315 |
| FKBP10 | Forward: ATGCCTTGAGTCAGATCCCC  Reverse: CCGCTCATTGACGCACATG | 205  291 |
