# Supplementary figures and images for: Integrative analysis of transcriptome complexity in pig granulosa cells by long-read isoform sequencing
Source: PeerJ. 2022 May 25;10:e13446. doi: 10.7717/peerj.13446 (PMC9147391; doi:10.7717/peerj.13446)

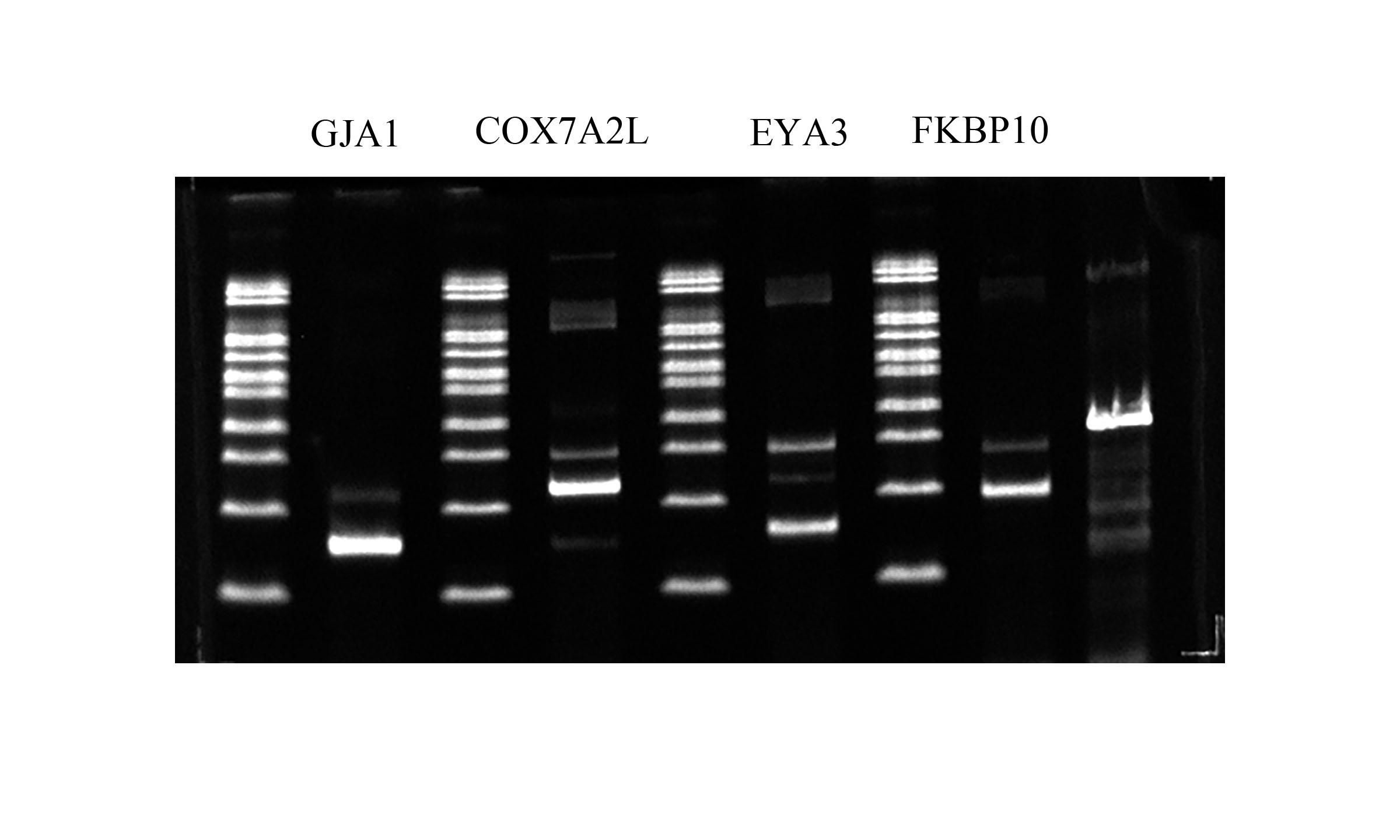

Supplement: Supplemental Information 7 [file peerj-10-13446-s007.jpg]
